# Colonic Atresia and Stenosis: Our Experience

**Published:** 2012-01-01

**Authors:** Bilal Mirza, Shahid Iqbal, Lubna Ijaz

**Affiliations:** Department of Paediatric Surgery, The Children's Hospital and the Institute of Child Health Lahore, Pakistan

**Keywords:** Colonic stenosis, Colonic atresia, Intestinal obstruction

## Abstract

Background: Colonic atresia and stenosis are rare entities. On average 1 case per year of colonic atresia is being seen in most of pediatric surgical centers and to date less than 10 cases of colonic stenosis have been reported.

Material and Methods: The medical record of patients of colonic atresia and stenosis managed during March 2006 to March 2010 was reviewed.

Results: A total of 15 patients of colonic atresia (11) and stenosis (4) were the study population. Four were ascending colon atresia, 2 at hepatic flexure and transverse colon each, and 1 at sigmoid colon. Two patients had multiple colonic atresias. One patient of ascending colon atresia also had pyloric atresia. In colonic stenosis population (two congenital and two secondary to necrotizing enterocolitis), two were transverse colon stenosis and two were sigmoid colon stenosis. The preoperative diagnosis was distal small bowel atresia in 11 patients. Colonic atresias were managed by colocolic anastomosis with covering ileostomy in 8 patients. The remaining 3 patients were managed by exteriorizing both ends of atresia. Colonic stenosis cases were managed by primary colocolic anastomosis in 1 patient and colocolic anastomosis under covering ileostomy in 3 patients. Three patients of colonic atresia succumbed postoperatively.

Conclusion: Colonic atresia and stenosis are rare entities. Associated alimentary tract malformations may result poor prognosis. Colonic atresia can safely be managed by colocolic anastomosis with covering ileostomy.

## INTRODUCTION

Colonic atresia and stenosis are the rare entities with an incidence (colonic atresia) of 1 in 20,000 live births and comprise about 1.8-15% of intestinal atresias. The colonic stenosis is even rarer and to date not more than 10 cases have been reported in the English literature [1,2]. We are presenting our experience of managing cases of colonic atresia and stenosis with reference to types, management and outcome.

## MATERIALS AND METHODS

The record of patients of colonic atresia and stenosis managed during March 2006 to March 2010 was reviewed and information regarding demography, diagnosis, operation and outcome was retrieved and analyzed.

## RESULTS

A total of 15 patients of colonic atresia (11) and stenosis (4) were the study population. In case of colonic atresia the mean age of presentation was 2.4 days (SD± 1.2 days) and male to female ratio was 6:5. In case of colonic stenosis one patient presented on 3rd day of life, 2 patients presented at 1.5 months of life, and one patient presented at 8 months of age (M:F 3:1). There was history of polyhydromnios in one patient.

The presentation was of neonatal intestinal obstruction in 12 cases and the preoperative diagnosis was distal small bowel atresia in 11 patients. Three other patients presented with off and on history of partial intestinal obstruction in terms of abdominal distension, passage of very small amount of stool and occasional vomiting. Of these 3, 2 patients had history of prematurity and development of abdominal distension and constipation within second week of life; nevertheless one patient had these symptoms since birth. X-ray abdomen erect was performed in every case that revealed multiple air fluid levels in each patient except in one patient where it revealed single gastric shadow indicative of pyloric atresia (Fig. 1). Ultrasound of the abdomen gave dilated bowel loops.

**Figure:**
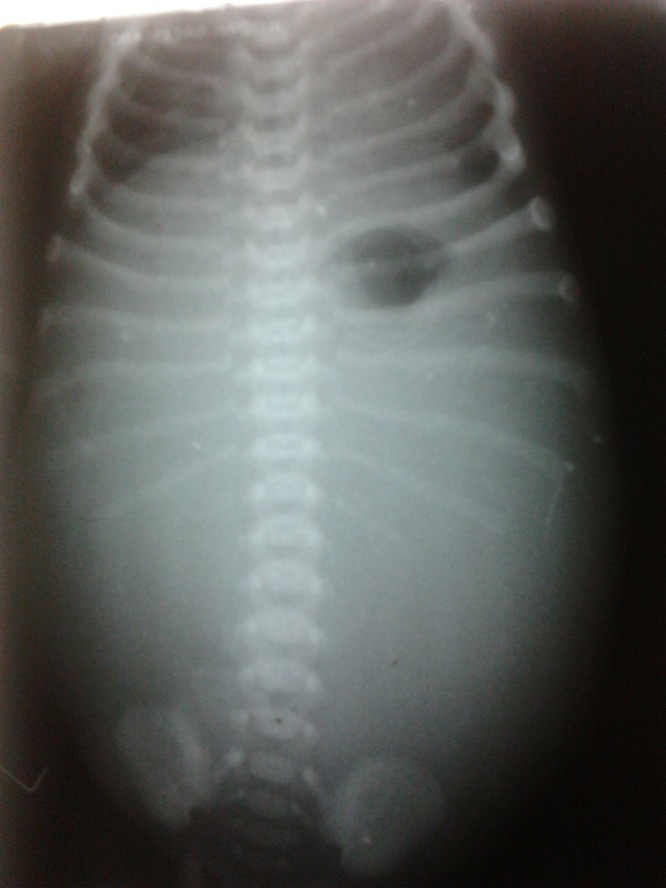
Figure 1: X ray showing single air shadow

At operation, four were ascending colon atresia (Fig. 2), 2 were hepatic flexure, 2 were transverse colon, and 1 sigmoid colon atresia (4 type I, and 5 type III variety). Two patients had multiple colonic atresias. One patient of ascending colon atresia also had pyloric atresia. In colonic stenosis population (two congenital and two secondary to necrotizing enterocolitis), two were transverse colon stenosis and two were sigmoid colon stenosis (Fig. 3,4). Colonic atresias were managed by colocolic anastomosis with covering ileostomy in 8 patients (in one patient pyloric atresia was managed by gastroduodenal anastomosis). The remaining 3 patients were managed by exteriorizing both ends of atresia. Colonic stenosis cases were managed by primary colo­colic anastomosis in 1 patient and colocolic anastomosis under covering ileostomy in 3 patients. Three patients of colonic atresia (one having associated pyloric atresia and two with multiple colonic atresias) succumbed post­operatively. In two of the patients of colonic atresia managed by exteriorization of ends of colon, the colostomy had been reversed. Whereas in all the surviving patients in whom colocolic anastomosis was performed under covering colostomy the ostomies have now being reversed.

**Figure:**
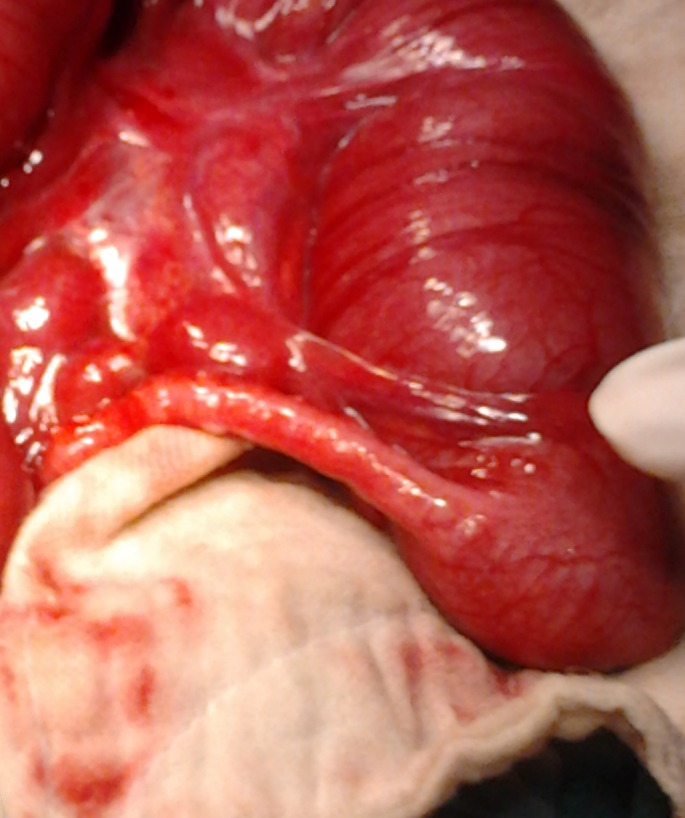
Figure 2: Ascending colon atresia

**Figure:**
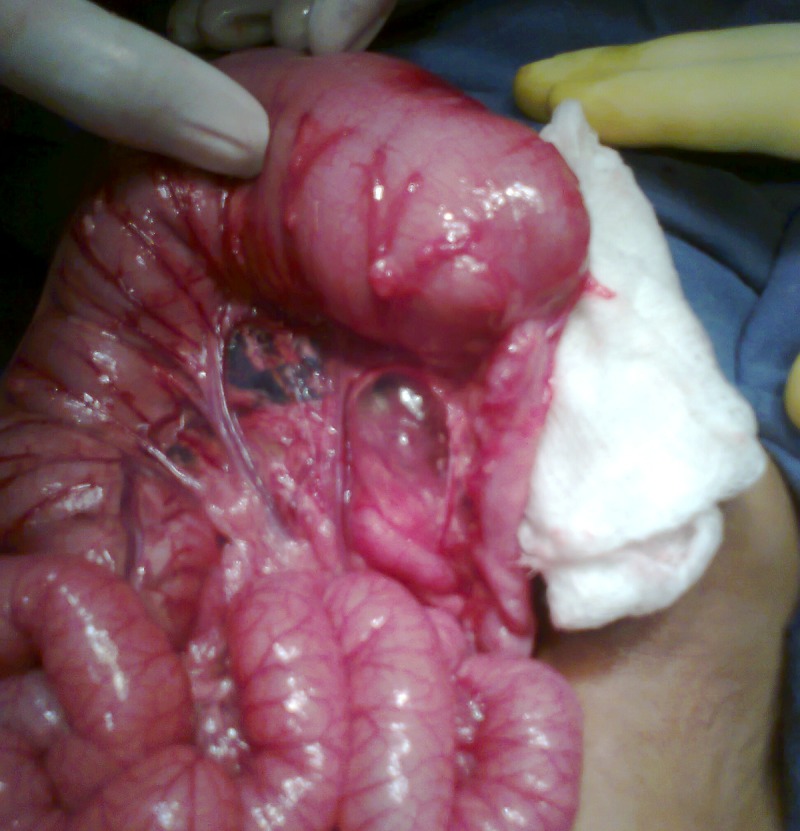
Figure 3: sigmoid colon stenosis in infant

**Figure:**
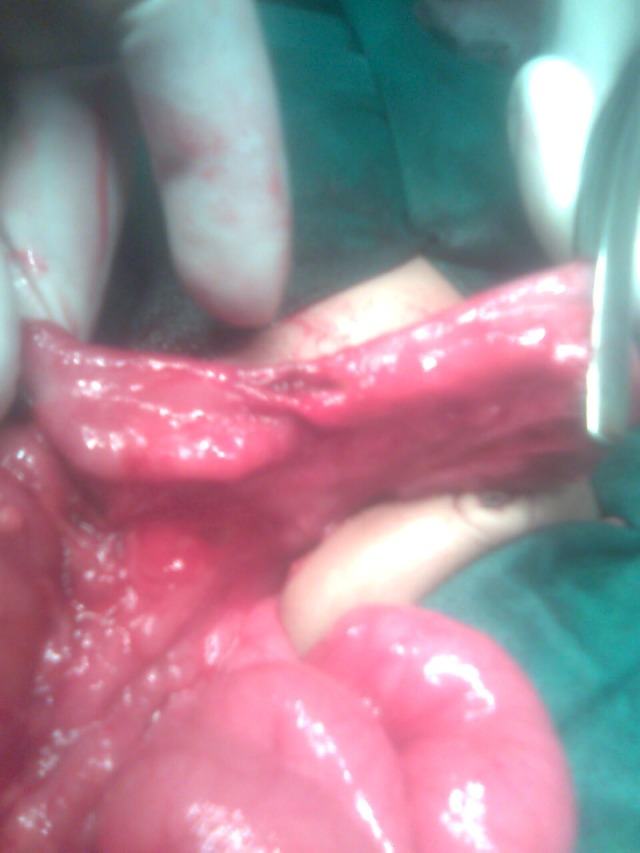
Figure 4: sigmoid colon stenosis in neonate

## DISCUSSION

In a series of 277 patients, over a period of 25 years, the incidence of colonic atresia was less than a case per year. Colonic stenosis is even rarer. The most commonly accepted theory of development of intestinal atresia and stenosis is based on mesenteric vascular accidents occurring during the course of fetal development. The other proposed theories are failure of recanalization, intestinal perforation, drugs and environmental factors. Vascular insufficiency is believed to cause colonic atresia and stenosis, nevertheless in one patient of ascending colon atresia associated with pyloric atresia the etiology seems to be failure of recanalization. Pujar et al reported first case of pyloric atresia associated with multiple colonic atresias however few other cases of pyloric atresia in association with multiple small as well as large bowel atresias have also been reported. Now we are reporting the second case of pyloric atresia in association with colonic atresia- the Pujar-Mirza syndrome [1-7].

Colonic stenosis can be congenital or acquired. In our series two patients (One presented on third day of life and other at 8m age) were of congenital colonic stenosis based on symptoms started since birth whereas two patients who were premature and developed presentation after second week of life considered secondary to necrotizing enterocolitis. The preoperative diagnosis of colonic atresia and stenosis can be made with the help of a contrast enema. Any newborn with a suspicion of intestinal atresia clinically and on plain radiograph should have a contrast enema to delineate the unused colon and ascertain any colonic atresia or stenosis. In case of colonic atresia the contrast will typically fill the lumen of distal unused colon, terminating at a point adjacent to the segment that is most distended with luminal air - the cutoff point. In case of colonic stenosis the contrast enema delineates an unused distal colon with a small caliber followed by a distended portion of colon, proximal to the stenosis [1,8,9].

The treatment option used in most of cases of left sided colonic atresia and stenosis is colocolic anastomosis with a covering enterostomy, and primary anastomosis is preferred by some authors in right colon lesions [2, 10-13]. We did primary colocolic anastomosis in case of colonic stenosis presented to us at 8 months of age, whereas, in other patients our preferred method remained colocolic anastomosis under covering enterostomy. Although we managed 3 patients of colonic atresia by exteriorizing the colonic ends but during reversal of the colostomy the huge difference of lumens in proximal and distal colonic ends posed difficulty in anastomoses. The outcome remained good in our patients having isolated colonic atresias and stenosis, but where it was associated with other alimentary tract atresias like pyloric atresia and multiple colonic atresias, the prognosis remained bad. These patients succumbed to sepsis in the postoperative period.

## CONCLUSION

Colonic atresia and stenosis are rare entities. Associated other alimentary tract malformations may indicate poor prognosis. Colonic atresia can safely be managed by colocolic anastomosis with covering ileostomy.

## Footnotes

**Source of Support:** Nil

**Conflict of Interest:** None declared

